# Unraveling the expression of differentially expressed proteins and enzymatic activity in response to *Phytophthora nicotianae* across different flue-cured tobacco cultivars

**DOI:** 10.1186/s12866-022-02531-z

**Published:** 2022-04-23

**Authors:** Ruifang Song, Yujiao Tan, Waqar Ahmed, Guisu Zhou, Zhengxiong Zhao

**Affiliations:** 1 College of Resources and Environment, Yunnan Agricultural University, Kunming, 650201 Yunnan China; 2 College of Tobacco Science, Yunnan Agricultural University, Kunming, 650201 Yunnan China; 3 State Key Laboratory for Conservation and Utilization of Bio-Resources in Yunnan, Yunnan Agricultural University, Kunming, 650201 Yunnan China; 4 Key Laboratory of Agro-Biodiversity and Pest Management of Ministry of Education, Yunnan Agricultural University, Kunming, 650201 Yunnan China

**Keywords:** *Phytophthora nicotianae*, Flue-cured tobacco, Defense-related enzymes, iTRAQ, Disease resistance

## Abstract

**Background:**

Black shank disease caused by *Phytophthora nicotianae* is a serious threat to flue-cured tobacco production. Whole-plant resistance is characterized by the expression of a number of pathogenesis-related proteins, genes, and the activity of different defense-related enzymes. In this study, we investigated the activity of defense-related enzymes and expression of differentially expressed proteins through the iTRAQ technique across two flue-cured tobacco cultivars, i.e., K326 and Hongda, in response to the black shank pathogen.

**Results:**

Results showed that the highest disease incidence was recorded in flue-cured tobacco cultivar Hongda compared with K326, which shows that Hongda is more susceptible to *P. nicotianae* than K326. A total of 4274 differentially expressed proteins were detected at 0 h and after 24 h, 72 h of post-inoculation with *P. nicotianae*. We found that 17 proteins induced after inoculation with *P. nicotianae*, including pathogenesis (5), photosynthesis (3), oxidative phosphorylation (6), tricarboxylic acid cycle (1), heat shock (1), and 14–3-3 (1) and were involved in the resistance of flue-cured tobacco against black shank disease. The expression of 5 pathogenesis-related proteins and the activities of defense-related enzymes (PPO, POD, SOD, and MDA) were significantly higher in the leaves of K326 than Hongda after inoculation with *P. nicotianae*.

**Conclusion:**

These results provide new molecular insights into flue-cured tobacco responses to *P. nicotianae.* It is concluded that differences in protein expressions and defense-related enzymes play an important role in developing resistance in flue-cured tobacco cultivars against black shank disease.

## Background

Black shank caused by an oomycete *Phytophthora nicotianae* is a destructive soilborne disease of tobacco (*Nicotiana tabacum* L) worldwide, including China [1]. Black shank disease was first reported in Java (Indonesia) in 1896 by Van Breda de Haan, and since then, it has been widespread around the globe. Tobacco crops are badly affected by this pathogen, which results in substantial yield losses [2]. *P. nicotianae* has a wide host range, infects 255 plant genera from 90 plant families, and causes crown rot, root rot, fruit rot, damping-off, leaf, and stem infection [3]. It can survive in the soil for a long period (4–6 years) even in the absence of a host plant and reproduces both sexually and asexually, having a reproduction cycle of 72 h [4, 5].

Tobacco crops can get affected by *P. nicotianae* at any growth stage from seedling to harvest, and it is difficult to control the disease [1]. In China, it is commonly known as Hei Jing Bing "Black Shank Disease" by the local farmers based on the typical disease symptoms [6]. The infected plants generally show symptoms of root and crown rot, yellowing, wilting, and necrosis of leaves. On cut stems, the signs of *P. nicotianae* are observed as white mycelium on the necrotic plate-like disks [7]. The average disease incidence of tobacco black shank is recorded between 10 to 20%, but it can reach up to 100% [8]. In severe outbreaks and prolonged drought periods, it occurs in epidemic form, resulting in plant death and destruction of the whole crop [9].

Soil environmental conditions such as rhizospheric microbial diversity, availability of nutrients and organic matter, soil texture, and soil pH are the main factors responsible for soilborne diseases [10]. Over the past few decades, important integrated disease management methods such as resistant cultivars [11], chemical pesticides (synthetic fungicides) [12], crop rotation, and soil replacement [13] have been adopted to control this disease. However, these methods have many disadvantages, such as the long-term application of synthetic fungicides may lead to resistance in the pathogen, affect soil microbial diversity, and are environmentally unfriendly [1, 14]. Therefore, microorganism-mediated biocontrol is considered a practical approach to mitigate soilborne diseases [15]. These microorganisms reduced the incidence of soilborne disease through the mechanism of competitive inhibition, production of antimicrobial compounds, induction of host resistance, and reshaping the soil microbiome's community structure [15, 16].

Cultivation and selection of disease-resistance cultivars play an important role to control tobacco black shank disease [11]. Many studies have proven that the incidence of black shank disease was higher in susceptible cultivars than resistance cultivars [17]. Upon pathogen attack, the plant defense system activates, which helps the plant to limit the invasion. During the defense activation the expression of certain disease-resistance changes significantly [18]. Disease-resistance proteins identify the proteins secreted by pathogens and trigger the immune responses to control pathogen infection [19]. Defense-related enzymes and compounds such as catalase (CAT), peroxidase (POD), superoxide dismutase (SOD), polyphenol oxidase (PPO), and malondialdehyde (MDA) are involved in the defense function and metabolic balance of plants [20, 21]. Proteomics is a practical approach to decipher an organism's physiological and pathological mechanisms [19].

For instance, the activities of defense-related enzymes POD, SOD, PPO, and PAL significantly increased in the stem and leaves of the patchouli plant after inoculation of *Ralstonia solanacearum* [22]. Many studies have proven that the content of MDA in tobacco leaves and enzyme activity such as SOD, POD, PPO, and CAT in tobacco plants increased after inoculation of black shank pathogen [23, 24]. Singh et al. (2021) used the iTRAQ technique to investigate the physiological role of pathogenesis-related proteins in two sugarcane varieties GT29 (smut-resistant) and Yacheng71-374 (smut-susceptible) with contrasting resistance to sugarcane smut. Results showed that after inoculation of *Sporisorium scitamineum,* the number of differentially expressed proteins was higher in GT29 than Yacheng71-374 [25]. Under normal conditions, plant defense-related enzymes and protein expression remained stable but when plants were exposed to biotic and abiotic stresses the protein expression changed significantly. However, it was found that defense-related enzymes and protein expression levels in different cultivars (resistant and susceptible) also differ. Therefore, enzymatic activity and protein expression in a healthy plant can be used as a reference index for screening of resistant cultivars.

Tobacco (*Nicotiana tabacum*) has been widely used as a model plant for in-depth molecular and biological studies. Scarce information is present on the molecular-based proteomic analysis and activities of defense-related enzymes in different flue-cured tobacco cultivars with contrasting resistance to blank shank disease. Therefore, the present study aims to fill this knowledge gap with more advanced techniques to explore the important mechanisms involved in two flue-cured tobacco cultivars with contrasting resistance against *P. nicotianae*. Differential responses of defense-related proteins and enzymatic activity help us to decipher the molecular mechanisms involved in flue-cured tobacco resistance against black shank pathogen.

## Methods

### Experimental site and design description

A greenhouse experiment was conducted during the growing season from March-August in 2017 at Technology Extension Station of Tobacco Company in Xundian (25°46' N, 102°55' E) County, Kunming City, Yunnan Province, China. Seedlings (45 days old) of two flue-cured tobacco cultivars, K326 and Hongda were transplanted in pots (40 × 35 cm) containing 13 kg of soil. To overcome nutrient deficiency fertilizers were applied twice as base fertilizer (N-P2O5-K2O = 8- 16- 22) 45 g/pot and top fertilizer (20 days after transplantation) (N- P2O5- K2O = 15–0-33) 25 g/pot [26]. Each pot was irrigated with 1000 ml/plant of water thrice a week to maintain moisture level. The greenhouse conditions were maintained as a day/night temperature (30/20℃) with a 14 h light/10 h dark photoperiod [27]. The experiment was conducted under a completely random design, and each treatment was repeated thrice with 45 plants/pots of each cultivar (K326 and Hongda) in each treatment. All integrated filed management practices and sample collection protocols were performed according to the National Standards of Tobacco Industry in China as described by Tang et al. [28].

### Pathogen strain, culture medium, and growth conditions

*Phytophthora nicotianae* used throughout this experiment was provided by Professor Guanghai Ji (State Key Laboratory for Conservation and Utilization of Bio-Resources in Yunnan), Yunnan Agricultural University, Kunming, China. Pure culture of *P. nicotianae* was prepared on oatmeal agar (OA) medium (Oatmeal 30 g/L; Agar 20 g/L; pH 7.0) and incubated at 27℃ for 7–10 days. Zoospores of *P. nicotianae* were prepared as described by Zhang et al. [29]; briefly, *P. nicotianae* was cultured in OA medium at 27℃ for three weeks. Then 10 ml/plate of 0.1% KNO_3_ solution was added, and plates were incubated at 27℃ for three days. After a sudden drop down to 4℃ for 30 min, the zoospore suspension was collected by filtering through gauze. The resulting zoospore suspension was centrifuged at 1200 rpm at 4℃ for 10 min. The supernatant was diluted with sterilized distilled water to a concentration of 1 × 10^6^ zoospores/ml using a hemacytometer.

### Inoculation of pathogen and sample collection

Zoospore suspension of *P. nicotianae* (1 × 10^6^ zoospores/ml) was prepared as described above, and inoculation was done 30 days after seedling transplantation at the 5–6 leaf stage. Before inoculation, each pot was irrigated with 1000 ml of water, and about 30 ml/plant of zoospores suspension was sprayed on the leaves [1]. Leaf samples were collected at 0 h (before inoculation) and after 24 h, 72 h of post-inoculation with *P. nicotianae*. The collected samples were stored in an ultra-low volume refrigerator at -80℃ for further study (analysis of defense-related anti-oxidative and proteomics study). Here, K-0, K-24, K-72 and HD-0, HD-24, HD-72 represent the samples collected from K326 and Hongda, respectively, at 0 h (before inoculation) and after 24 h, 72 h of post-inoculation.

### Analysis of defense-related enzymes

#### Polyphenol oxidase activity

Polyphenol oxidase activity was determined with the catechol colorimetric method by measuring the absorbance rate of quinone formation at OD_525 nm_ using a spectrophotometer (PharmaSpec 1700) as described by Sikora et al. [30]. An increase in absorbance rate of 0.01 min^−1^ for quinone formation was taken as one unit of enzyme activity. The sample contained 0.3 ml of crude enzyme solution, two ml of phosphate buffer (0.05 mmol/ml and pH 7.0), and one ml of catechol (0.1 mol/ml).

#### Peroxidase activity

Oxidation of guaiacol is the rate-determining step for peroxidase activity. Peroxidase activity was determined by the guaiacol method using the methodology of Zhang and Shao [31]. Briefly, 3.9 ml of the reaction solution was added (28 µl of 30% H_2_O_2_, 50 ml of 0.1 mol/L phosphate buffer (pH 6.0), and 19 µl of guaiacol) to a 10-ml test tube and placed in a water bath at 25℃ for 5 min. Then, 0.1 ml of crude enzyme solution was added to the test tube, mixed thoroughly, and an increase in the absorbance rate of 0.01 min^−1^ at OD_470 nm_ using a spectrophotometer (PharmaSpec 1700) was taken as one unit for enzyme activity.

#### Superoxide dismutase activity

Superoxide dismutase activity was evaluated by measuring its ability to inhibit photochemical reduction of nitro blue tetrazolium using the methodology of Kong et al. [32]. The reaction mixture (2.725 ml) contained (26 mM methionine, 1 μM EDTA, 20 μM riboflavin, 50 mM phosphate buffer (pH 7.8), and 750 μM nitro blue tetrazolium). After adding crude enzyme solution (25 μl) and distilled water (250 μl) into the reaction mixture, the reaction was allowed to run at 25–35 °C for 20 min under a 4000-lx fluorescent lamp. The absorbance of the reaction mixture was measured at OD_560 nm_ using a spectrophotometer (PharmaSpec 1700). The 50% nitro blue tetrazolium photochemical reduction inhibition was taken as an enzyme activity unit.

#### Malondialdehyde activity

Malondialdehyde contents were determined using the thiobarbituric acid colorimetric method as described by Gao and Zhang [33]. Briefly, 0.2 g of samples were ground on ice in 10% trichloroacetic acid to make a homogenized mixture. The mixture was then added to a 3 ml solution (0.5% thiobarbituric acid + 10% trichloroacetic acid) and incubated at 90℃ for 15 min in a water bath. After this, the suspension was cooled down at room temperature and centrifuged at 10,000 rpm for 10 min at 4℃. The supernatant was collected and light absorbance was measured at OD_450 nm_, OD_532 nm_, and OD_650 nm_ using a spectrophotometer (PharmaSpec 1700). Malondialdehyde contents were measured using following formula: MDA contents (nmol/g) = [6.45 × (A532-A650)—0.56 × A450] × VT/FW/VS. Here: VT is the volume of extraction solution; VS is the volume of test solution; FW is the weight of the sample.

### Proteins extraction and iTRAQ assay

#### Proteins extraction and quantification

Proteins extraction, quantification, and digestion were done as previously described by Xie et al. [22]. Total protein was extracted from the frozen dried leaf samples, ground in liquid nitrogen, and extracted with lysis buffer (2 M thiourea, 7 M urea, 40 mM Tris–HCl, and 4% 3-[(3-Cholamidopropyl) dimethylammonio]-1-propanesulfonate (CHAPS), pH 8.5) supplemented with two mM ethylenediaminetetraacetic acid (EDTA) and one mM phenylmethane sulfonyl fluoride (PMSF). After 5 min of vigorous vortexing 10 mM dithiothreitol (DTT) was added to the suspension mixture. The suspension was sonicated at 200 W for 15 min and centrifuged at 15000 rpm for 20 min. The supernatant was collected and mixed with a 5 × volume of chilled acetone containing 10% (v/v) trichloroacetic acid (1:4, v/v) and incubated overnight at − 20℃. Proteins in the supernatant were collected and stored at − 80℃ for further analysis.

#### iTRAQ labeling and fractionation

Samples were labeled with iTRAQ 8-plex Multiplex Reagent Kit (AB Sciex U.K.) by following the manufacturer's instructions. All labeled samples were mixed in equal volume and fractionated using a high-performance liquid chromatography (HPLC) system (Thermo Dionex Ultimate 3000 BioRS).

#### LC–MS/MS proteomic analysis

LC–MS/MS analysis was performed in buffer A (0.1% formic acid and 2% acetonitrile), and the final concentration of peptides was adjusted to 0.5 μg·μL^−1^. From each sample, ten μL supernatant was loaded into a two cm C18 trap column in an LC-20 CE nano HPLC (Shimadzu, Kyoto, Japan) via an auto-sampler. Samples were loaded at a rate of 8 μl/min for 4 min. The following 41 min gradient was then run at 300 nL/min: 5% to 35% B (0.1% formic acid 98% and acetonitrile), 5 min linear gradient to 80%, 80% B for 5 min, and a one min decrease to 5%. The peptides were subjected to nano-electrospray ionization followed by MS/MS via a Q-Exactive mass spectrometer (Thermo Fisher Scientific, San Jose, CA, USA).

#### Bioinformatics analysis

The whole experiment was performed in triplicate with 3–5 analytical replications in each treatment. Proteins identification and quantitative analysis (false-discovery rate (FDR) ≤ 0.05) were performed using the Mascot 2.3.02 search engine against the *Nicotiana tabacum* database (http://www.ncbi.nlm.nih.gov/protein?term) and Proteome Discoverer™ Software (Thermo), respectively. The relevant parameters used for screening of differentially expressed proteins are shown in Table 1. Proteins with fold changes ≥ 1.2 or ≤ 0.833 and Q-values < 0.05 in at least two replicate/treatment were considered as differentially abundant proteins [34]. To determine the biological process, molecular process, and cellular component, blast2go software (V4.5 pipeline) was used for GO annotation and compared with the gene ontology (GO) database (http://geneontology.org/) for the differences in protein components. Kyoto Encyclopedia of Genes and Genomes (KEGG) database (www.kegg.jp/kegg/kegg1.html) was used to classify and group the identified proteins [35]. Data were analyzed statistically using a *t-*test to determine differences at a 5% significance level with Microsoft Excel 2013 and SPSS version 22.0 (SPSS, Chicago, IL, USA) [36]. All figures were processed and analyzed using Adobe Illustrator CS5 (Adobe Systems Inc., San Francisco, CA, USA).

**Table 1:** Selection of relevant parameters in appraisal for screening of differentially expressed protein

Name of parameters	Experimental options
Enzyme	Trypsin
Max Missed Cleavages	2
Fixed modifications	Carbamidomethyl (C), iTRAQ 8plex (N-term), iTRAQ 8plex (K)
Variable modifications	Oxidation (M), iTRAQ 8plex (Y)
Peptide Mass Tolerance	± 20 mg/Kg
Fragment Mass Tolerance	0.1 Da
Database	uniprot_*Nicotiana tabacum*
Peptide FDR	≤ 0.01
T-test	*p* ≤ 0.05
Fold change	≥ 1.2 or ≤ 0.83

#### Disease incidence

Disease incidence was recorded once a week after post-inoculation till the end of the experiment. Disease incidence was graded according to GB/T 23222–2008 (National Standardization Management Committee of China, 2009) using a disease rating scale as follows: 0; healthy plants, 1; one-third of the leaves were wilted and diseased spot on stem does not exceed one-third of the stem, 3; half of the leaves were wilted and diseased spot exceeded half of the stem, 5; two-third of leaves were wilted and necrosis of older leaves, 7; all leaves were wilted and necrosis of more than half of leaves, 9; death of whole plant [37]. Disease incidence (DI) was calculated using the following formula: DI (%) = (Number of infected plants/Total investigated plants) × 100.

## Results

### Analysis of disease incidence

**Fig. 1:**
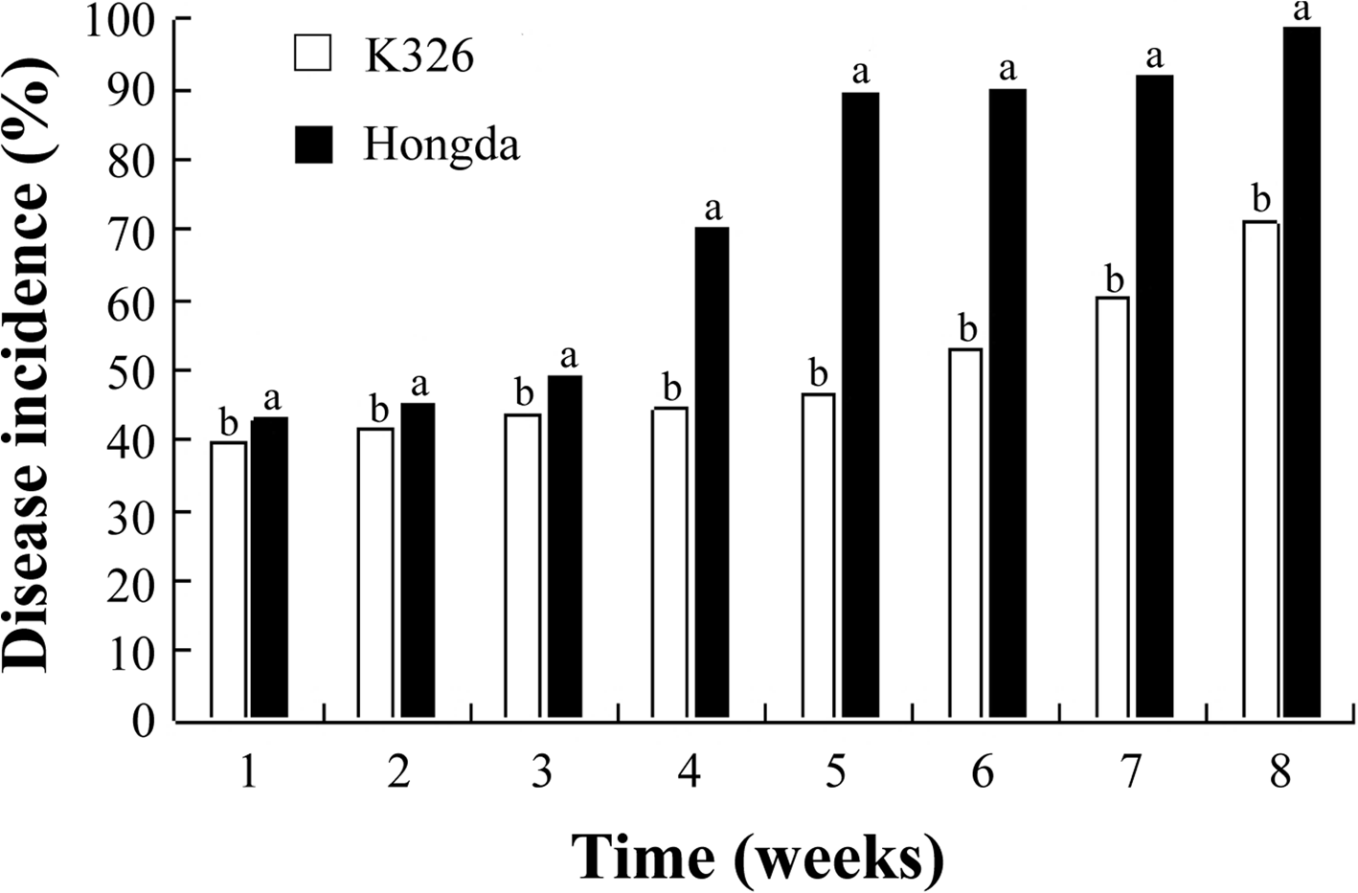
Disease incidence (%) in two flue-cured tobacco cultivars (K326 and Hongda). Significant difference among flue-cured tobacco cultivars is shown by different small letters on the bars, according to Duncan's multiple range test at *p* < 0.05

It was found that different flue-cured tobacco cultivars show different level of resistance against black shank disease. Severe environmental conditions like continuous high temperature and high humidity were provided through the experiment after the inoculation of *P. nicotianae*. In the early stage of infection, no significant difference was observed in disease incidence between K326 and Hongda. But as time passed, a significant difference was observed between K326 and Hongda in disease occurrence. In the case of Hongda, the disease incidence was reached up to 100% after the 8^th^ week of post-inoculation, whereas K326 showed more resistance against black shank disease than Hongda (Fig. 1).

### Effect of *P. nicotianae* on the activity of defense-related enzymes in different flue-cured tobacco cultivars

**Fig. 2:**
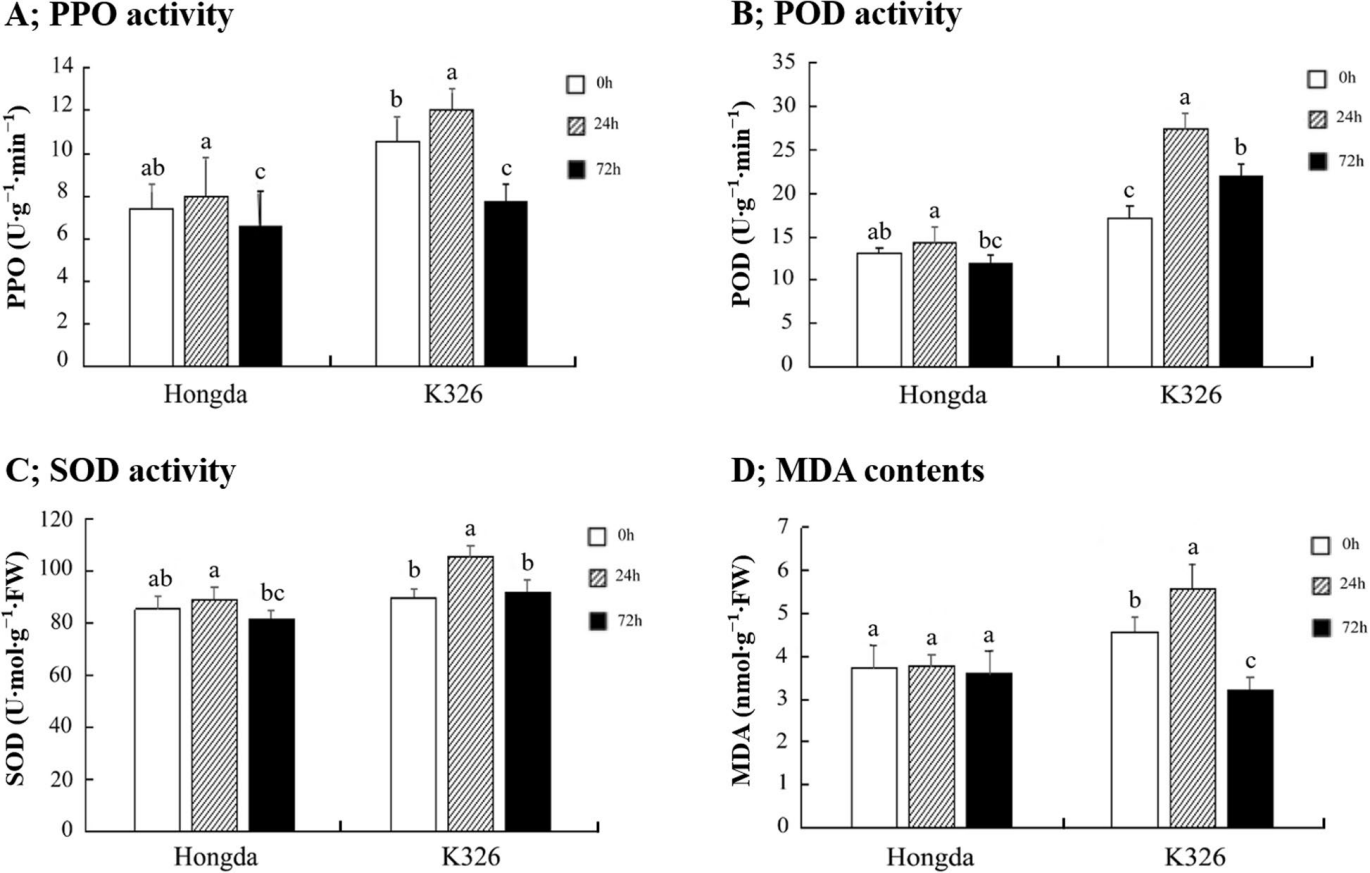
Activities of defense-related enzymes across different flue-cured tobacco cultivars in response to black shank pathogen. PPO activity (**A**), POD activity (**B**), SOD activity (**C**), and MDA contents (**D**). Error bars represent the standard error of means (± SEM, *n* = 3). Different small letters on error bars indicate a significant difference according to Duncan's multiple range test at *p* < 0.05

The activity of different defense-related enzymes (PPO, POD, and SOD) and MDA contents were examined in leaves of two flue-cured tobacco cultivars K326 and Hongda at 0 h, and after 24 h and 72 h of post-inoculation with *P. nicotianae*. Results revealed that the activity of PPO, POD, and SOD enzymes and MDA contents were significantly higher in K326 than Hongda (Fig. 2). However, the activity of PPO, POD, and SOD enzymes and MDA contents in both flue-cured cultivars K326 and Hongda initially increased and then decreased after 24 h and 72 h of post-inoculation with *P. nicotianae*, respectively.

### Proteins identification and profiling

**Fig. 3:**
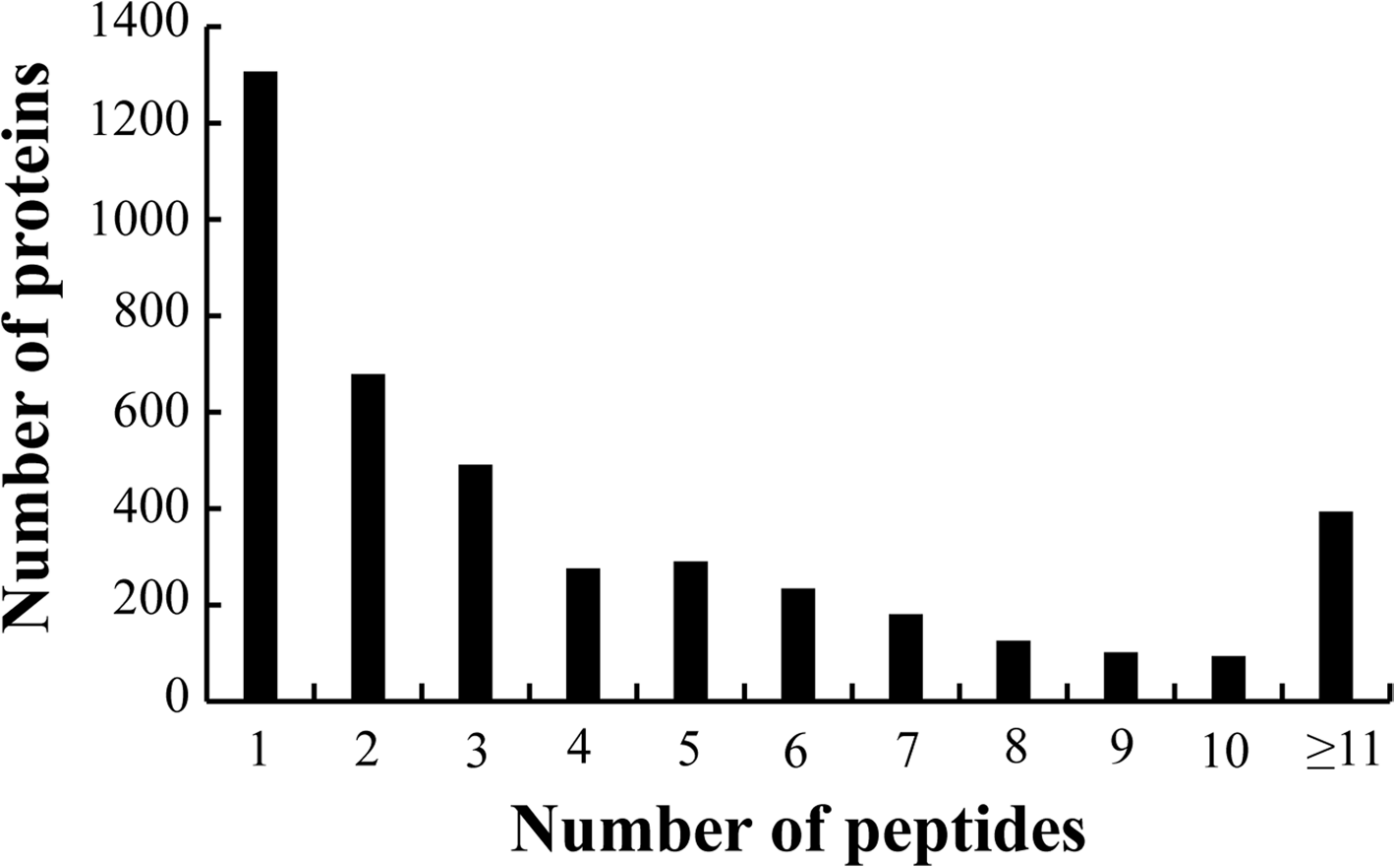
Proteins identification and profiling for differently expressed proteins. Data are shown as the standard error of means (± SEM, *n* = 3) at *p* < 0.05

In this study, a total of 405,918 MS/MS spectra were confirmed using the iTRAQ LC–MS/MS technique; among them, 51,812 spectra matched with known protein spectra in the database. In addition, 14,146 peptides, 9,918 unique peptides, and 4,274 differentially expressed proteins were identified, of which 69.4% of proteins contained more than two peptides, as shown in Fig. 3. Proteins were screened based on a fold-change value ≥ 1.2 or ≤ 0.833 and a *p-*value < 0.05. Differential abundant protein screening was performed in three replicates and differential abundant proteins that were appeared at least twice in three replicates were selected for subsequent analysis.

### Analysis of differentially expressed proteins

**Fig. 4:**
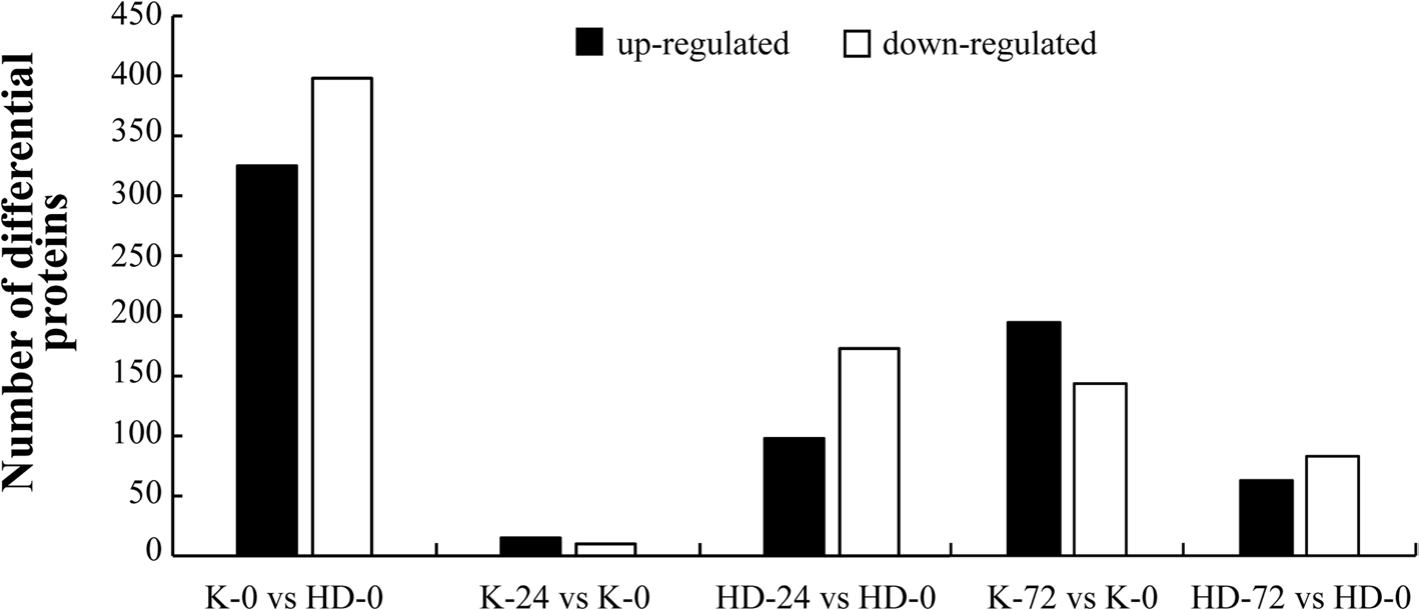
Identification of differentially expressed protein in group-wise comparison across different flue-cured tobacco cultivars after specific hours of post-inoculation with *Phytophthora nicotianae*. Data are shown as the standard error of means (± SEM, *n* = 3) at *p* < 0.05. Here: K; K326, HD; Hongda, 0; before inoculation at 0 h, and 24, 72; after 24 h and 72 h of post-inoculation

The number of differentially expressed proteins obtained by statistical analysis is shown in Fig. 4. In this study, a total of 723 differentially expressed proteins (325 up-regulated and 398 down-regulated) were found in a group vise comparison between K-0 vs HD-0, indicating that selected cultivars K326 and Hongda are different from each other based on their genetics. Similarly, a total of 35 (15 up-regulated and 10 down-regulated), 271 (98 up-regulated and 173 down-regulated), 318 (183 up-regulated and 135 down-regulated), and 137 (59 up-regulated and 78 down-regulated) differentially expressed proteins were identified between K-24 vs K-0, HD-24 vs HD-0, K-72 vs K-0, and HD-72 vs HD-0, respectively.

### Bioinformatics analysis for differentially expressed proteins

**Fig. 5:**
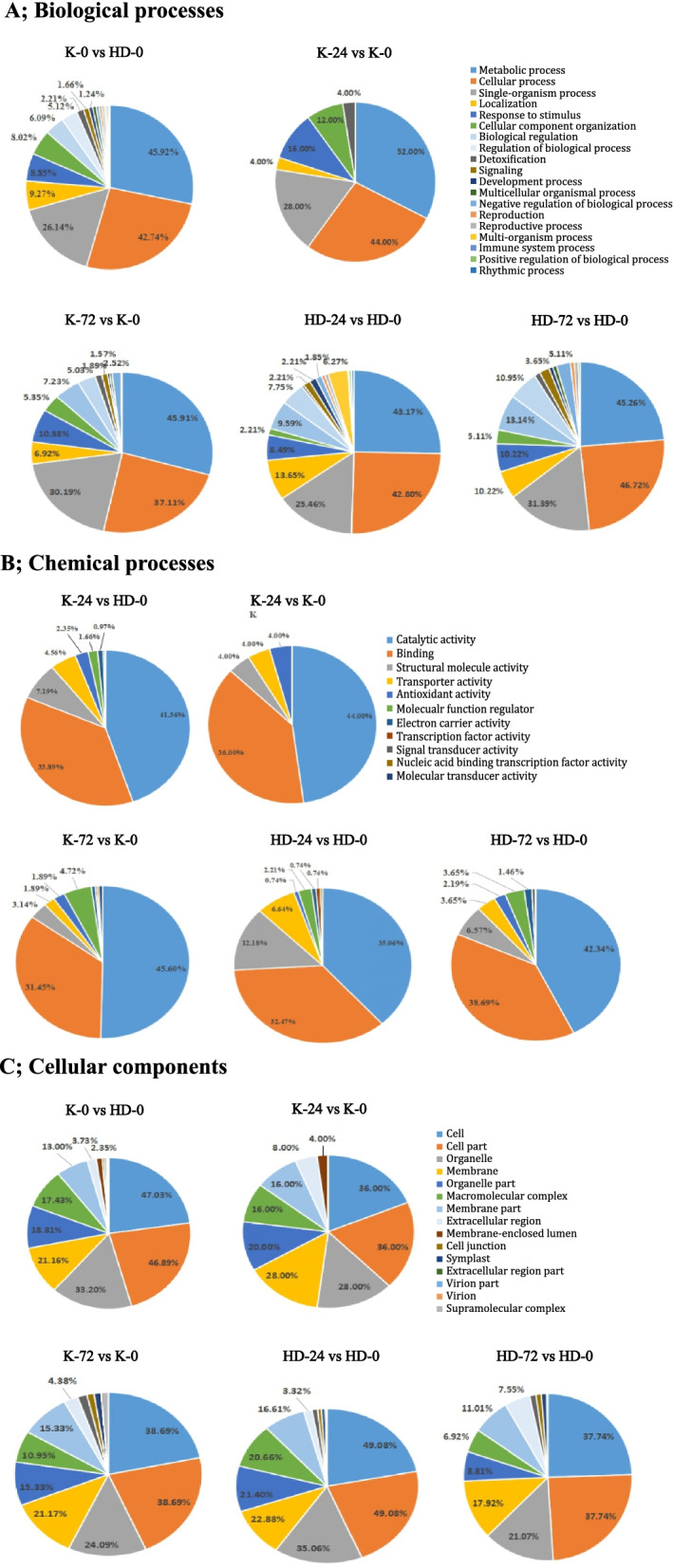
GO classification analysis for differentially expressed proteins in group-wise comparison across different flue-cured tobacco cultivars after specific hours of post-inoculation with black shank pathogen. All identified proteins (4274 in total) were categorized according to Biological processes (**A**), Molecular processes (**B**), and Cellular components (**C**). Here: K; K326, HD; Hongda, 0; before inoculation at 0 h, and 24, 72; after 24 h and 72 h of post-inoculation

GO classification analysis was performed for differentially expressed proteins between 5 different groups (K-0 vs HD-0, K-24 vs K-0, HD-24 vs HD-0, K-72 vs K-0, and HD-72 vs HD-0) as shown in Fig. 5 (A; biological processes, B; molecular processes, C; cellular components). Results showed that differential proteins were mainly involved in cellular processes, metabolic processes, single-cell processes, biological regulation, and response to a stimulus. Proteins related to molecular functions are mostly binding proteins and catalytic activities (enzymes). As cell components, proteins are mainly involved in cells, cell parts, organelle, and membrane.

### Screening of proteins related to induction of black shank pathogen

**Fig. 6:**
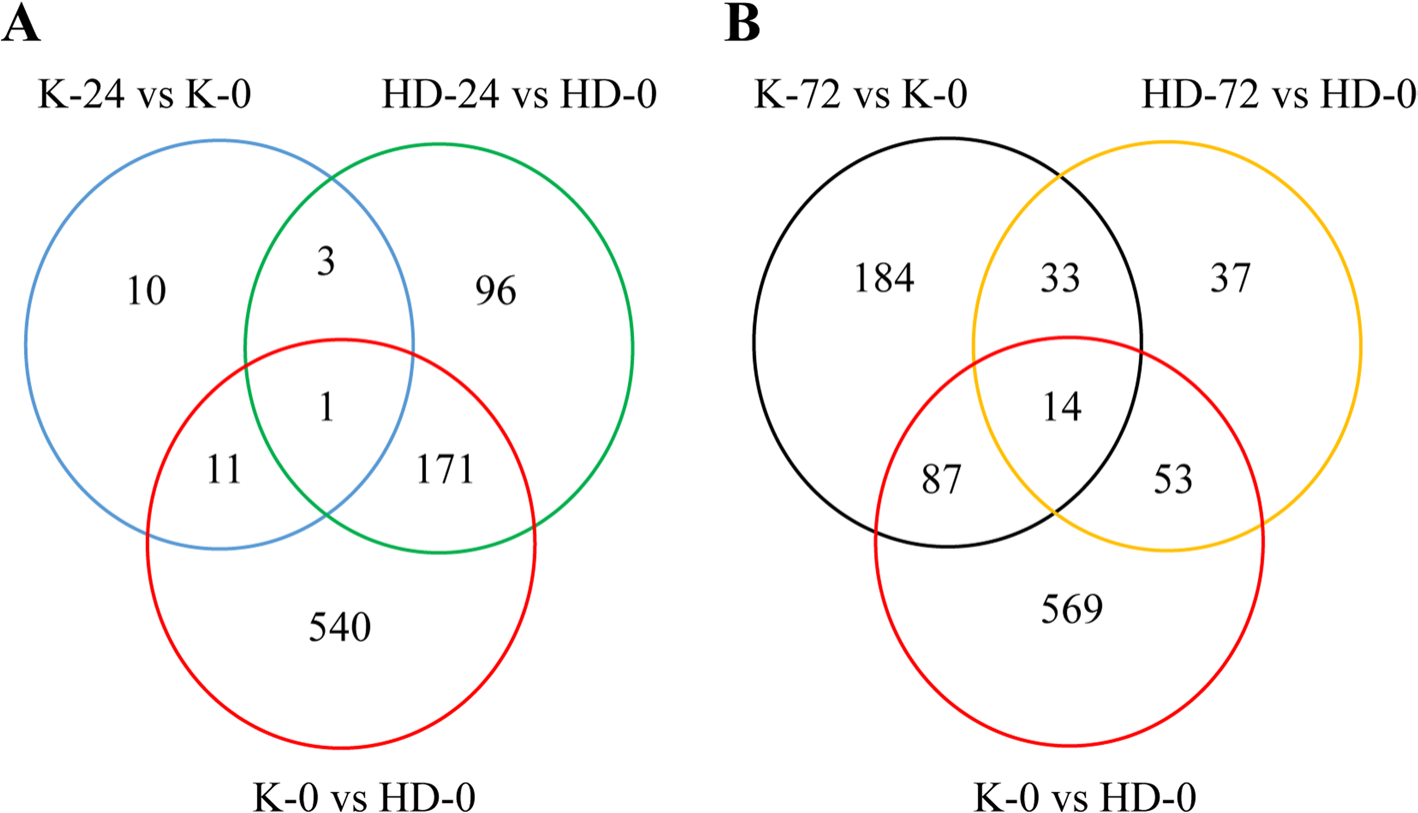
Venn diagram for differentially expressed proteins in flue-cured tobacco cultivars (K326 and Hongda) after inoculation of black shank pathogen. Here: 24 h of post-inoculation (**A**) and 72 h of post-inoculation (**B**)

To further investigate the difference in response of K326 and Hongda to black shank infection, differentially expressed proteins of two flue-cured tobacco cultivars were analyzed after 24 h and 72 h of post-inoculation with *P. nicotianae* (Fig. 6). Results revealed that after 24 h of post-inoculation, K326 showed ten unique proteins, while Hongda showed 96 unique proteins. However, 11 proteins in K326 and 171 in Hongda were induced after 24 h of post-inoculation. Four different proteins were found in two flue-cured cultivars after 24 h of post-inoculation, among which one showed an opposite expression trend and three showed the same trend (two up-regulated and one down-regulated) (Fig. 6A).

Unique proteins in K326 increased to 184, while specific proteins in Hongda decreased to 37 after 72 h of post-inoculation. However, 87 proteins in K326 and 53 in Hongda were induced after 72 h of post-inoculation. There were 47 differentially expressed proteins in two flue-cured cultivars, among which five showed the opposite expressions, and 42 showed the same expression trends (27 up-regulated and 15 down-regulated) (Fig. 6B). It was found that 17 proteins were involved in the resistance of flue-cured tobacco to black shank after 0 h (before inoculation) and 24 h, 72 h of post-inoculation (Table 2). By identifying and screening the above 17 differential proteins, five pathogenies-related proteins, three photosynthesis-related proteins, six proteins involved in oxidative phosphorylation, one protein involved in the tricarboxylic acid cycle, one heat shock protein, and one 14–3-3 protein were found. These specific proteins may be an important factor in determining flue-cured tobacco resistance to the black shank disease.

**Table 2:** Resistant related proteins in two flue-cured cultivars at 0 h (before inoculation), and after 24 h, 72 h of post-inoculation with *Phytophthora nicotianae*

Protein ID	**Accession**	**K-0 VS HD-0**	**K-24 VS K-0**	**HD-24 VS HD-0**	**K-72 VS K-0**	**HD-72 VS HD-0**
0A1S3XSS2	Pathogenesis-related leaf protein 4-like	0.6625	0.9102	0.6998	1.3644	1.1072
P29062	Pathogenesis-related protein PR-4A	0.5105	0.7673	0.4720	1.8383	0.7037
Q6LBM4	Pathogenesis-related protein 4B (Fragment)	0.7108	0.6381	0.5936	1.8101	1.0473
A0A1S4CVY1	Pathogenesis-related protein STH-2-like	1.1043	––-	––-	2.1105	1.4748
A0A1S3XZH5	Pathogenesis-related protein STH-2-like	1.0119	––-	––-	1.6837	1.4321
A0A1S3XWY6	Photosystem I reaction center subunit XI	0.6481	1.0642	0.5081	1.0404	0.9257
P69556	Photosystem II protein D1	0.8037	0.9760	0.6045	1.0282	0.9767
A0A140G1P7	ATP synthase subunit alpha	0.7465	1.2009	0.9603	1.1116	0.9027
A0A1S3YW39	Soluble inorganic pyrophosphatase 6, Hloroplastic-like	1.4556	1.2485	1.1330	0.9451	1.0870
A0A1S3YLE3	NADH dehydrogenase [ubiquinone] 1 alpha subcomplex subunit 2-like	0.5768	1.2399	0.7302	1.0675	0.8569
A0A1S4CUD1	Peroxidase	1.2987	1.6497	1.0476	1.0065	1.2897
A0A1S3XUV5	Endoplasmin homolog	1.3066	1.0823	1.0842	0.9301	1.0085
A0A1S3ZQP6	Polyphenol oxidase E, chloroplastic-like isoform X1	1.4512	0.8258	1.0259	1.0036	0.9547
Q75ZE5	14–3-3 a-1 protein	0.7620	1.0484	1.0965	1.0613	1.1955
A0A1S4CJX9	Glutathione S-transferase T1-like	1.2084	1.2104	1.1031	0.9818	1.0574
A0A1S3XQB7	Peroxidase	1.7804	1.8519	1.1507	––-	––-
A0A1S3XFZ4	Dihydrolipoamide acetyltransferase component of pyruvate dehydrogenase complex	0.7619	1.3047	0.9921	––-	––-

Here: K; K326, HD; Hongda, 0; 0 h (before inoculation), and 24; 24 h, 72; 72 h of post inoculation with *Phytophthora nicotianae*, respectively.

## Discussion

### Activities of defense-related enzymes across different flue-cured tobacco cultivars in response to black shank pathogen

Induced systemic resistance and systemic acquired resistance of plants play an important role in disease development after a pathogen attack. Studies have proven that the activities of defense-related enzymes in plants remain stable during normal growth and change when exposed to biotic and abiotic stresses [38]. An increase in the activity of PPO enzyme can enhance the plant's resistance to biotic and abiotic stresses [39]. Similarly, POD and SOD are known to be as important scavenging enzymes of reactive oxygen species (ROS) in plants [40]. Peroxidase can catalyze hydrogen peroxide (which is toxic to plants) into non-toxic water and oxygen. Simultaneously, SOD removes superoxide anion free radicals produced by plants under stress and protects cells from damage [41, 42]. We observed that PPO, POD, and SOD enzymes expression were different among different flue-cured tobacco cultivars. After being infected with black shank pathogen, the activities of PPO, POD, and SOD enzymes increased in both cultivars K326 and Hongda, but enzyme activity was found higher in K326.

Malondialdehyde is a final product of membrane lipid peroxidation in plants under stress and is an important biochemical index to reflect the degree of membrane damage. When plants are under stress, higher malondialdehyde contents in leaves indicate a higher degree of membrane and cell damage [42, 43]. In our study, we found that the contents of MDA in the leaves of K326 were increased initially and then decreased significantly after the inoculation of *P. nicotianae*. Although the varying trend in the MDA contents in Hongda remained consistent, and no significant difference was found compared to control (0 h before inoculation). Our study demonstrates that the activities of PPO, POD, and SOD enzymes and MDA contents in the leaves of K326 increased significantly compared with Hongda after the inoculation of *P. nicotianae*. This may be related to systemic acquired resistance of K326 to black shank pathogen, while Hongda is more susceptible to black shank disease than K326.

### Differentially expressed proteins induced by flue-cured tobacco cultivars in response to *P. nicotianae*

In this study, a total of 4274 differentially expressed proteins were found in the leaves of K326 and Hongda via iTRAQ technique after the inoculation of *P. nicotianae*. Among them, 723, 25, 271, 318, and 137 differentially expressed proteins were identified in a group-wise comparison between K-0 vs HD-0, K-24 vs K-0, HD-24 vs HD-0, K-72 vs K-0, and HD-72 vs HD-0, respectively. Further analysis of differentially expressed proteins between K326 and Hongda revealed that protein composition and contents in Hongda changed earlier than K326, which is similar to the findings of Parker et al. [44]. The resistance of K326 to black shank disease mainly manifests in a degree of damage to the photosynthetic system, cell oxidation balance, lignin synthesis capacity, and the induction strength of potential resistant genes. Preliminary, we found that twelve proteins were induced by the black shank pathogen. The expression of one D1 protein and one PPO protein decreased in K326 after pathogen inoculation, and the rest of the proteins showed an upward trend. The expression of two proteins involved in photosynthesis and one protein involved in oxidative phosphorylation was down-regulated in Hongda after the inoculation of *P. nicotianae*. So, we conclude that changes in the expression of differential proteins may be closely related to the genetic resistance of flue-cured tobacco cultivars to the black shank pathogen.

### Proteins associated with photosynthesis

It is well documented that photosynthetic system is very sensitive to external biotic and abiotic stresses. The changes in the photosynthetic system are the most fundamental characteristic of plant response to external stresses [45]. Studies have proven that whenever plants are subjected to external biotic or abiotic stresses, the expression of most of the genes related to photosynthesis pathway change significantly [45]. Among differentially expressed proteins, it was found that three proteins were involved in the photosynthesis of tobacco plants. As the core component of photosynthetic system PSII, rapid turnover of Photosystem II, protein D1 is the main prerequisite for PSII to function. However, light-driven electron transfer is necessary for the turnover of D1 protein function to repair PSII [46]. Our study demonstrates that D1 protein was down-regulated after the inoculation with *P. nicotianae*. The proteins related to Photosystem I reaction center subunit XI as a core component of chloroplasts were also down-regulated in Hongda after inoculation of black shank pathogen. Another photosynthesis-related protein, the ATP synthase-α subunit, was up-regulated in K326 after the inoculation of black shank pathogen. These results revealed that the black shank pathogen has a specific role in promoting photosynthesis in resistant cultivars.

### Tricarboxylic acid (TCA) related proteins

The host's defense response to pathogens is an active and energy-consuming process. In the main catabolic pathway of organisms, TCA is related to the mutual conversion of three major substances and acts as an important metabolic pathway that provides a large amount of free energy to organisms [47]. Through analysis of differentially expressed proteins, we found that one protein is involved in the TCA cycle, and the expression of acetyltransaminase-pyruvate dehydrogenase complex was up-regulated in K326 after inoculation of *P. nicotianae*. Results showed that the TCA pathway is activated in K326 after inoculation of black shank pathogen, thereby producing enough intermediate substances and energy to overcome pathogen infection [48]. This change may be related to the higher disease resistance of K326 towards *P. nicotianae* than Hongda.

### Oxidative phosphorylation and lignin synthesis related proteins

It has been found that ROS outbreak is one of the early disease-resistant responses of the host against pathogen and ROS production is a key signal in plant defense response [49]. Reactive oxygen species play an important role in plant defense response, but excessive accumulation of ROS can cause damage to normal cells. In order to reduce the contents of ROS in cells, plant cells have evolved a complex and refined elimination mechanism such as; antioxidants ascorbic acid, glutathione, and antioxidant enzymes [50]. Studies have proven that SOD, POD, and other enzyme activities in tobacco plants increase after black shank infection [23]. In this study, POD, SOD, and PPO contents were significantly increased in K326 after inoculation of *P. nicotianae*. It shows that POD, PPO, and SOD play a vital role in maintaining the balance of reactive oxygen species in cells and cell membranes' stability. Lignin is a complex phenolic resin polymer and is important for maintaining plant cell structure and resistance against pathogen infection [51]. Studies have proven that POD also plays a vital role in the synthesis of plant lignin [52, 53]. Two differentially expressed proteins screened were identified as POD, and their expression was found up-regulated in both tobacco cultivars (K326 and Hongda) after the inoculation of *P. nicotianae*. The contents of POD were found significantly higher in K326 than Hongda after inoculation of *P. nicotianae*. Similarly, the disease incidence was recorded minimum in K326 compared with Hongda, indicating that K326 activates its immune system against pathogen invasion.

### Pathogenesis-related proteins

Pathogenesis-related proteins (PRPs) are a class of proteins produced by plants in a pathological or pathologically related environment. Pathogen infection, abiotic stress, allergic reactions, and system acquired resistance can stimulate the accumulation of PRPs [54]. Therefore, the production and activity of PRPs play an important role in plant's resistance to biotic and abiotic stress [54]. In this study, we found that the expression of PR-4 family protein was down-regulated in K326 and the expression of STH-2 family protein was up-regulated compared with Hongda. After 24 h of post-inoculation, expression levels of three PR-4 family proteins in K326 and Hongda were down-regulated. However, after 72 h of post-inoculation compared with 0 h of post-inoculation, the expression of three PR-4 family proteins and two STH-2 family proteins in the K326 were up-regulated. The expression of three PR-4 family proteins and two STH-2 family proteins in the Hongda was down-regulated and up-regulated, respectively. In addition, the expression of other resistance-related proteins also changed significantly in K326 and Hongda after inoculation of *P. nicotianae*.

14–3-3 protein is involved in the regulation of primary metabolism, ion transport, intracellular transport, enzyme activation, and gene expression [55]. Many studies have proven that the 14–3-3 protein interacts with chaperonin Hsp70 to form a guide complex that mediates thylakoid proteins import [56, 57]. Yan et al. (2004) transformed the *Arabidopsis thaliana* GF14λ gene encoding 14–3-3 protein into cotton. They found that the transgenic cotton wilting rate was low under drought conditions and photosynthesis efficiency was higher than wild-type cotton [58]. In K326, we found that the expression of 14–3-3a-1 protein was up-regulated after inoculation of *P. nicotianae*, which improves K326 resistance to *P. nicotianae*. Our results are in accordance with the study of Lee et al., who reported that 14–3-3 protein in the nucleus of sweet pepper is involved in transcriptional regulation against TMV infection [59].

## Conclusion

In the light of obtained results, we conclude that the iTRAQ-based study expands our knowledge of defense-related enzymes and pathogenesis-related proteins in two flue-cured cultivars K326 and Hongda, after inoculation of *P. nicotianae*. A total of 4274 differentially abundant proteins were identified using the iTRAQ-based quantitative proteomic approach. Functional analysis of these differentially abundant proteins revealed that proteins were involved in photosynthesis, TCA cycle, oxidative phosphorylation, lignin synthesis, and PRPs. Enzymes such as PPO, POD, SOD, and MDA play an important role in the defense mechanism of flue-cured tobacco plants during black shank pathogen infection. Energy- and ROS-related biological processes and pathways were actively regulated in leave of K326 when exposed to *P. nicotianae*. These results advance our knowledge to understand the molecular changes involved in different flue-cured tobacco cultivars with contrasting resistance to black shank disease. Although proteomic analysis is a long-term goal, further studies should be done on protein signatures and transcriptomic analysis to screen the resistant cultivars against back shank pathogen.

## Data Availability

The datasets generated and analyzed during the current study are available in the iProX (https://www.iprox.cn/page/HMV006.html) having Accession Number IPX0003938000.
